# Aluminium Electrodeposition from Ionic Liquid: Effect of Deposition Temperature and Sonication †

**DOI:** 10.3390/ma9090719

**Published:** 2016-08-24

**Authors:** Enrico Berretti, Andrea Giaccherini, Stefano M. Martinuzzi, Massimo Innocenti, Thomas J.S. Schubert, Frank M. Stiemke, Stefano Caporali

**Affiliations:** 1 Dipartimento di Chimica, Università di Firenze, Sesto Fiorentino 50019, Italy; enrico.berretti@unifi.it (E.B.); andrea.giaccherini@unifi.it (A.G.); stefano.martinuzzi@unifi.it (S.M.M.); m.innocenti@unifi.it (M.I.); 2 IoLiTec Ionic Liquids Technologies GmbH, Heilbronn 74080, Germany; schubert@iolitec.de (T.J.S.S.); stiemke@iolitec.de (F.M.S.); 3 Consorzio Interuniversitario Nazionale Per la Scienza e Tecnologia dei Materiali (INSTM) and Istituto Sistemi Complessi (ISC), Sesto Fiorentino 50019, Italy

**Keywords:** ionic liquids, aluminium, electrodeposition, surface morphology, sonication, corrosion resistance, texture

## Abstract

Since their discovery, ionic liquids (ILs) have attracted a wide interest for their potential use as a medium for many chemical processes, in particular electrochemistry. As electrochemical media they allow the electrodeposition of elements that are impossible to reduce in aqueous media. We have investigated the electrodeposition of aluminium from 1-butyl-3-methyl-imidazolium chloride ((Bmim)Cl)/AlCl_3_ (40/60 mol %) as concerns the effect of deposition parameters on the quality of the deposits. Thick (20 μm) aluminium coatings were electrodeposited on brass substrates at different temperatures and mixing conditions (mechanical stirring and sonication). These coatings were investigated by means of scanning electron microscope, roughness measurements, and X-ray diffraction to assess the morphology and the phase composition. Finally, electrochemical corrosion tests were carried out with the intent to correlate the deposition parameters to the anti-corrosion properties.

## 1. Introduction

**Figure 1:**
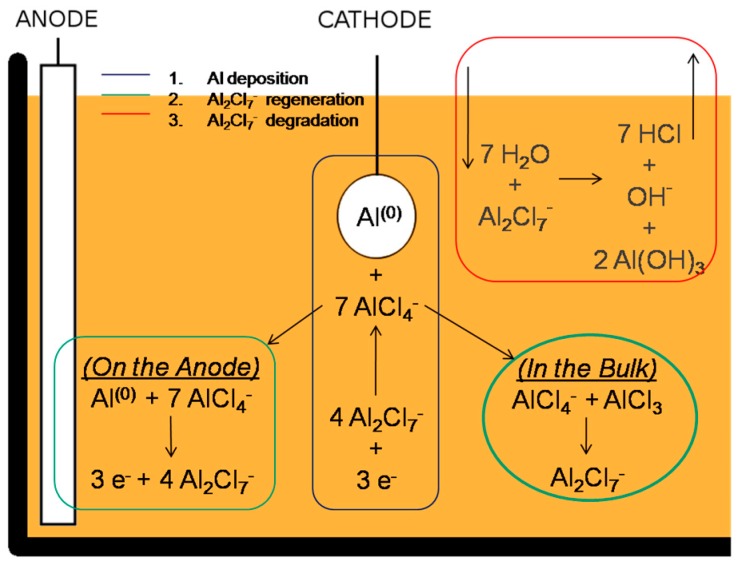
Scheme of the Al-electroplating process from chloroaluminated ionic liquids (ILs). Al is reduced at the cathode while at the anode the electroactive species is regenerated by aluminium dissolution and reaction with AlCl_4_^−^. The red box depicts the parasite reaction with moisture.

Since its invention, the Hall-Héroult process [1] has been the conventional process for the production of metallic aluminium. It is based upon the electroreduction of alumina in a cryolite melt by using graphite electrodes. The high temperature required (in the order of 900 °C) and the emission of toxic gaseous by-products, such as fluorine and carbon monoxide, rendered the research for greener and safer alternatives highly attractive [2]. The introduction of Ionic Liquids (ILs) (namely room temperature molten salts) as electrochemical media for the aluminium electrodeposition offered a very promising route to succeed in this goal. Since the first pioneeristic studies in the 1990s [3], a large number of investigations successfully reported the lab-scale electrodeposition of aluminium at room or nearly room temperature. Different types of ionic liquids [4,5,6,7,8,9] and additives [10,11] were proposed to achieve smooth and thick aluminium coatings. However, with the aim of the industrialization of the Al-plating process, the use of additive-free chloroaluminate ILs, seems to constitute the better compromise between the quality of the deposit and the durability of the electroplating bath. Beside the handling difficulties, mainly due to the vigorous reaction with atmospheric moisture, their high aluminium content, reduced viscosity, and remarkable electrochemical stability allow for a high deposition rate, in the order of 10 μm·h^−1^, and a long working time without the need to replenish chemicals. The scheme of the Al-electroplating process is depicted in Figure 1. 

The Al-coatings obtained at lab scale by means of this technology were successfully employed as protective layers for structural materials such as carbon steel [10,11,12], magnesium alloys [13], and light weight alloys [14], just to name a few. The anticorrosion properties of the Al-coatings are based upon the formation of a dense passivation layer composed by aluminium oxide that prevents further corrosive action towards the metal beneath. Therefore, the anticorrosion ability requires the formation of a homogeneous, crack-free alumina layer. 

In order to obtain high quality Al-coatings, the study of the effect of deposition parameters such as temperature and mixing conditions on the deposition rate, the crystalline structure, the surface morphology, and roughness is, therefore, mandatory. Electrodeposition in stirred solution has the advantage of reducing the thickness of the diffusion layer, thereby improving the deposition rate and the homogeneity of the deposit. However, even though some studies investigated the effect of temperature [12,15,16], very little work was reported on the effect of solution stirring [17,18] and, to the best of our knowledge, no one has addressed the effect of sonication so far. 

For these reasons, with an aim to determine the better operative conditions for the industrialization of the Al-plating process, we investigated the effects of temperature and mixing conditions, both alone and combined, on the electrodeposition of Al from the ionic liquid (Bmim)Cl/AlCl_3_ (40/60 mol %). Finally, the deposits were characterized by scanning electron microscopy (SEM) investigation, roughness measurement, X-ray diffraction, and electrochemical corrosion tests to assess the conditions more suited to achieving smooth, homogeneous, and thick aluminium coatings. 

## 2. Materials and Methods

### 2.1. Chemicals

The electroplating bath was constituted by 1-butyl-3-methyl-imidazolium chloride ((Bmim)Cl)/AlCl_3_ (40/60 mol %). It was supplied by IoLiTec Ionic Liquids Technologies GmbH (Heilbronn, Germany) and used as received without further purification.

### 2.2. Electrode Materials

12 mm diameter brass (Cu 63%, Zn 37%) tokens were employed for the study of temperature and mixing effect. Larger tokens (25 mm diameter) were used to study the combined effects. The tokens were grinded with SiC paper down to 1200 grit and polished with diamond suspension (Metadi 3 micron, Buehler, Uzwil, Switzerland), rinsed with tap water and sonicated in acetone (technical grade, VWR Chemicals, Leuven, Belgium) for 5 min. Before the electrodeposition they were rinsed again in tap water and soaked in HCl 1 M for 3 min, rinsed in distilled water, sonicated in acetone for 5 min and then dried under N_2_. Inside the nitrogen filled glove box, prior to the start of electrodeposition, the working electrodes were electrochemically cleaned in the electroplating bath by applying a positive (oxidative) current (2.5 mA·cm^−2^) for 30 s. 

### 2.3. Deposition Parameters

**Figure 2:**
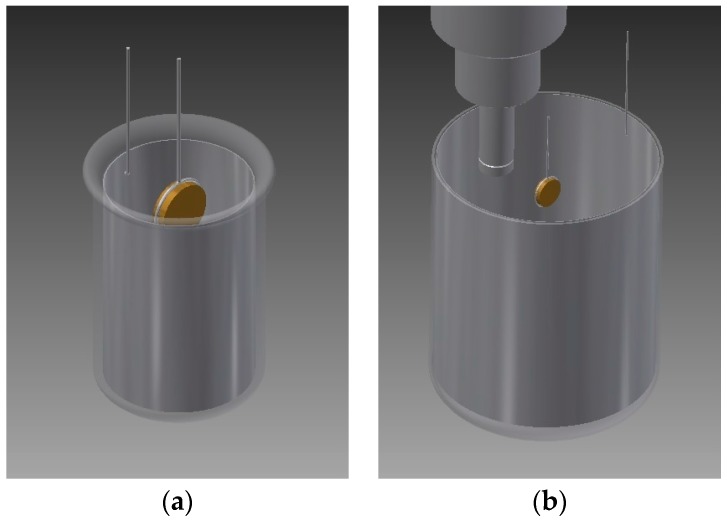
Scheme of experimental set-ups used for temperature (**a**) and sonication (**b**) experiments.

The plating process was carried out inside a nitrogen filled glove box (Iteco mod 10A, Castelbolognese, Italy), with water content below 20 ppm. The deposits were produced in current controlled (galvanostatic) conditions at 10 mA·cm^−2^ using a potentiostat (Model 7050 by Amel s.r.l., Milan, Italy), a circular anode constituted by a pure aluminium foil (Goodfellow, Huntingdon, UK, 99.0%) and brass tokens as cathodes. No reference electrode was used in this study, since the galvanostatic method requires only the control of the charge. The obtained potential vs. time curves refer to the absolute potential difference applied to the electrodes. Two different deposition set-ups were used with the same geometry but different anode/cathode surface ratio (see Figure 2a,b). Depositions were performed in quiet (no motion) or under mixing conditions by means of mechanical stirring (magnetic bar, 320 rpm) or sonication (Qsonica Sonicator Q500, Newtown, CT, USA, 500 W 20 kHz operating at 30% of maximum power). In the sonication experiments, the horn was placed on the cathode side as depicted in Figure 2b.

### 2.4. Characterization of the Deposits 

The morphology of the coatings was investigated by Scanning Electron Microscopy (S-2300, Hitachi, Tokyo, Japan) operating at 20 kV, while the average roughness was measured using a Hommel Tester W55 (Teplice, Czech Republic). The roughness measurements were obtained carrying out five different and independent tests in randomly chosen areas of the sample scanning 4.8 mm of surface at 0.2 mm·s^−1^ of scan rate. The parameters employed were λc = 0.8 mm and λc/λs = 300 using a filter ISO 11562(MI). Different roughness parameters were calculated (see Appendix A) and the obtained values are summarized in Table 1 and Table 2.

### 2.5. Process Yield

Since the IL viscosity is much larger with respect to the aqueous solutions (thus limiting the mass transport of the electroactive species), the formation of dendritic deposits is favoured, especially when mild operative conditions (low temperature, no mixing) and high current densities are employed. If dendritic coatings are formed, the detachment of parts of the deposit during the rinsing operations cannot be neglected. To take into account this mass loss, process yield was calculated as the percent ratio between the effective mass of the obtained deposit and the theoretical mass calculated from the Faraday law (Equation (1)).

(1) $$Yield=\frac{weightedMass}{theoreticalMass}\times 100$$

From the potentials reached in our experiments, there is no evidence that parasitic reactions can lower the cathodic efficiency of the deposition below a 100% yield. Therefore, considering the aluminium layer homogeneously distributed, the yield provides information about the thickness of the deposit. 

### 2.6. XRD Analysis

The phase determination of the coatings was carried out through X-ray diffraction (XRD), performed using a XRD Bruker D8 Advance powder diffractometer (Bruker AXS GmbH, Karlsruhe, Germany) employing Cu Kα (1.54187 Å) radiation in the 2θ range 36°–80°, applying a step size of 0.022° 2θ and a step counting time of 0.27 s. XRD spectra deconvolution was performed by means of the single line profile analysis [19], involving the fitting of the peaks with two Voigt functions (one for the K_α1_ and for K_α2_). We are aware that for the complete assessment of the strain and size of the crystallites the Warren-Averbach method had to be used. However, the accurate assessment of these parameters requires tens of independent peaks and it is beyond the aim of this work. Still, the use of Voigt functions (convolution of Lorentzian and Gaussian functions) allows us to assess the average strain of the crystallites, related to the Gaussian width, and the crystallite size, related to the Lorentzian width, in a simple and reliable way as shown by several authors [19,20,21]. In such a way, information on the effect of the experimental conditions on the strain and size of the crystals as well as the texture coefficient were determined. The texture coefficient T(hkl) for the (*hkl*) plane is defined in Equation (2):

(2) $$T\left(hkl\right)=\frac{A\left(hkl\right)/A_{0}\left(hkl\right)}{\sum_{hkl}A\left(hkl\right)/A_{0}\left(hkl\right)}$$

where A(hkl) is the experimental area under the Voigt curve of the (hkl) plane and $A_{0}\left(hkl\right)$ is the respective theoretical area simulated by using the program Mercury and Wycoff structural parameters [22,23,24].

### 2.7 Electrochemical Corrosion Test

The electrochemical corrosion tests were performed using a corrosion cell from Princeton Applied Research (Flat cell KO235, Oak Ridge, TN, USA) in aerated 3.5% NaCl (>99.5% from Merck, Darmstadt, Germany) solution at room temperature (20–23 °C). The potentiostat (Princeton Applied Research model 2273, Oak Ridge, TN, USA) was controlled by PowerSuite 2.58 software (Princeton Applied Research, Oak Ridge, TN, USA). The classical three electrode set-up was employed using a platinum grid as counter electrode and a standard calomel electrode (SCE) reference electrode separated from the solution with an ions conducting glass frit, the working electrode surface was always 1.0 cm^2^. The tests were performed on the aluminized samples without further treatment. Every sample was kept in the saline solution for at least 16 h in order to allow the stabilization of the open circuit potential (OCP); then, potentiodynamic (PD) experiments were recorded starting from −0.250 V with respect to the OCP at the scan rate of 0.4 mV·s^−1^.

## 3. Results

### 3.1. Effect of Temperature

**Figure 3:**
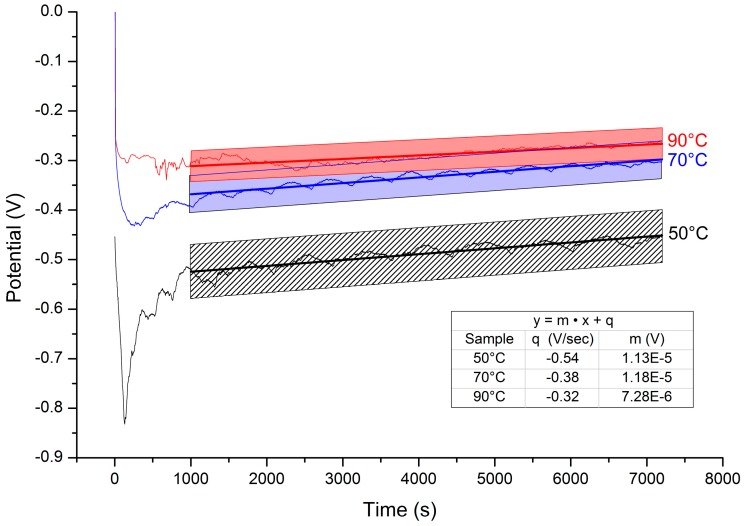
Potential-time curves obtained at different temperatures. Galvanostatic deposition 10 mA·cm^−2^, deposition time 2 h. The inset depicts the results of data linear fitting. The slope of the curves decreases with increasing temperature.

Even though the effect of temperature on the morphology of the Al-coatings has been previously reported for this IL up to 55 °C [14], we extended the investigation at higher temperatures. In this set of experiments, while maintaining quiet the electroplating bath, the Al deposition was performed at three different temperatures: 50 °C, 70 °C and 90 °C. During the deposition the potential-time curves (Figure 3) were recorded in order to extrapolate the induction time (the time needed to reach a stable deposition potential) and the equilibrium potential. The last one provides direct insight about the overpotential needed to obtain the Al-reduction and, therefore, the energy consumed. 

In accordance with previous investigations in similar ionic liquids [14], higher temperature favours the electroreduction process. Increasing temperature reduces the induction time, promotes higher process yield, and requires less negative potentials. It is noteworthy that the curves are characterized by smaller slopes indicating, on the whole, a lower surface increase as a consequence of the formation of smoother deposits. This qualitative observation was supported by more quantitative roughness measurements. The data summarized in Table 1 show that the surface roughness decreased and more homogeneous deposits (lower relative error in the roughness measurement) were obtained as a function of temperature increase. 

**Figure 4:**
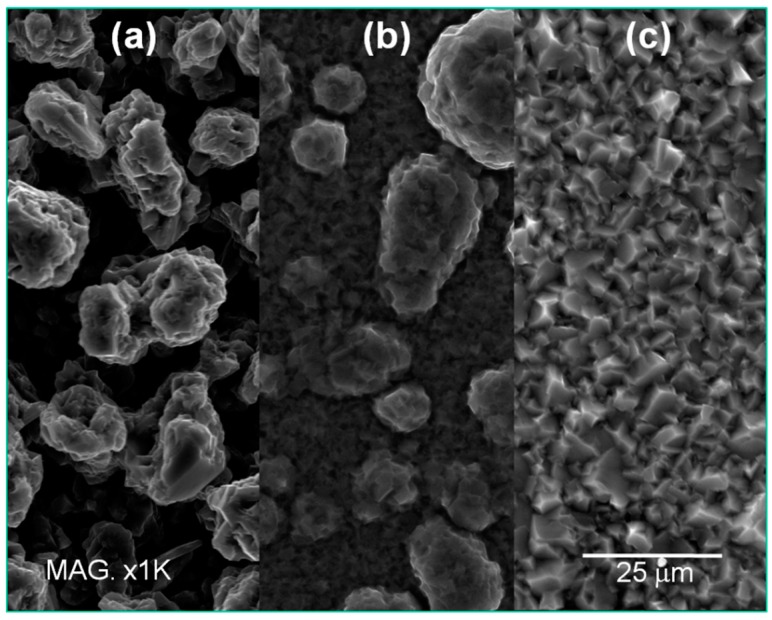
Scanning electron microscopy (SEM) images of the samples obtained at different temperatures: (**a**) 50 °C; (**b**) 70 °C; and (**c**) 90 °C. Deposition time was 2 h.

SEM investigation (Figure 4) strengthen this trend evidencing the change of morphology from pinnacle type deposits at 50 °C to homogeneous crystalline structure at 90 °C. 

Unfortunately, repeated experiments demonstrate that for temperatures higher than 70 °C the ILs undergo very rapid degradation. This impairs the use of higher temperatures for industrial and applicative purposes. The recommended maximum temperature can be considered 60 °C. All the following experiments were carried out at temperatures not exceeding 60 °C. 

### 3.2. Effect of Sonication

By using the larger set-up (Figure 2b) necessary to immerse the sonication horn in the electroplating bath, two series of samples were produced at 20 °C employing different sonication duty cycles. The first one, namely mild sonication, consisted of 1 s on (30% power) for every 10 s off; the second, stronger sonication, consisted in a 1 s on (30% power) for every 1 s off. We refer to these conditions as “Sonication 1 to 10” and “Sonication 1 to 1” respectively. Quiet depositions were also carried out for comparison. 

**Figure 5:**
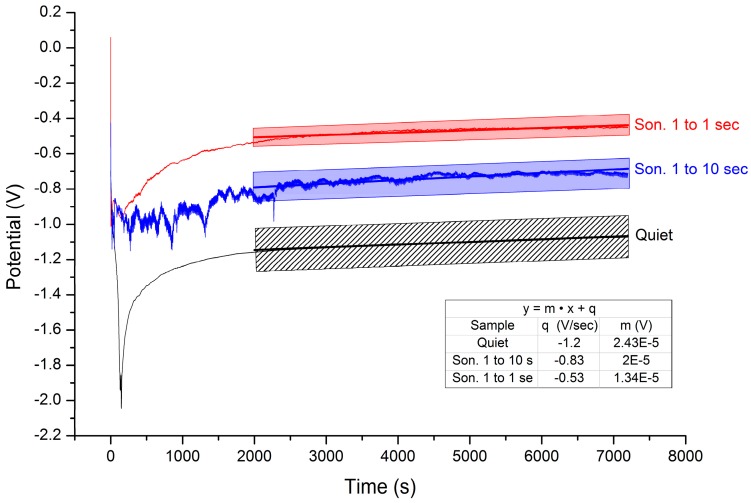
Potential-time curves obtained at different sonication levels. Galvanostatic deposition 10 mA·cm^−2^, deposition time 2 h. The inset depicts the results of data linear fitting; shadow areas represent the confidence interval.

**Figure 6:**
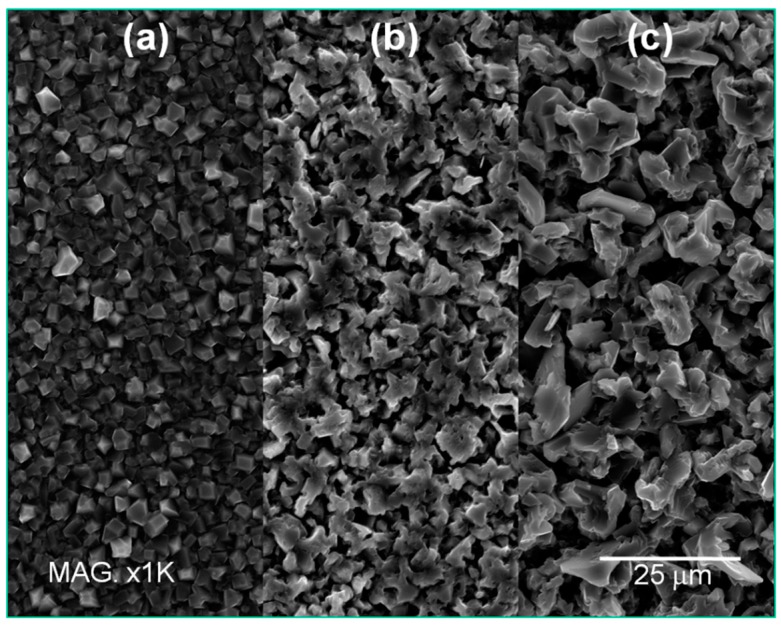
SEM images of the samples obtained using different sonication: (**a**) Quiet solution; (**b**) Sonication 1 to 10; and (**c**) Sonication 1 to 1.

The potential-time curves depicted in Figure 5 show long induction time and the serious effect of sonication on the equilibrium potential; stronger sonication leads to less negative deposition potentials and, in turn, an easier deposition process. On the other side, the roughness measurements summarized in Table 2 depict an opposite trend with respect to the temperature series indicating, on the whole, the increase of surface roughness as a function of sonication. This counter intuitive behaviour is explained by SEM investigation (Figure 6). Even if the nucleation and growth mechanism remain unchanged, increasing the sonication power leads to the formation of larger crystals that account for the increased surface roughness. It is also worth noting that by using sonication a 100% yield is achieved. On the other hand, the deposit obtained at 20 °C without sonication presents a massive deposit loss (yield about 60%, Table 2). That is reasonably due to the formation of dendrites that were removed during sample rinsing. As a consequence of the reduced thickness, the surface roughness decreases. 

#### Corrosion Tests

**Figure 7:**
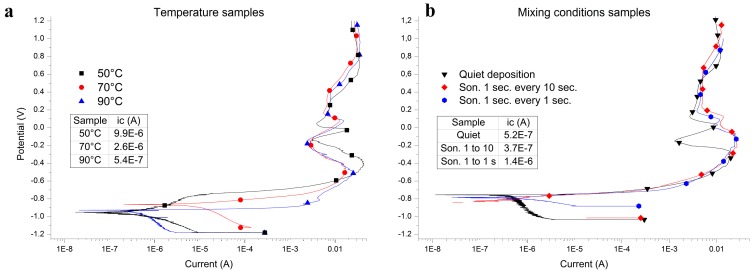
Potentiodynamic curves in 3.5% NaCl aerated aqueous solution on samples obtained at different temperatures (**a**) and mixing conditions (**b**). Scan rate 0.4 mV·s^−1^. The inset depicts the corrosion current values.

The Al-coatings obtained at different temperatures and mixing conditions were investigated to assess their anticorrosion properties in 3.5% NaCl aerated aqueous solution. Figure 7 shows the potentiodynamic curves relative to the samples obtained at different temperatures (a) and mixing conditions (b). There were no substantial variations among the free corrosion potentials. Only a modest decrease in the corrosion current (i_c_) is detectable in the temperature series. That is reasonably due to the decrease in sample roughness and, therefore, the reduction of the active surface. On the whole, all the curves but one present very similar anodic branches thereby confirming a similar corrosion mechanism. The different curve shape displayed by the sample obtained at room temperature in quiet conditions is reasonably due to the lower thickness, about 12 μm accordingly to a 60% yield, with respect to the 20 μm of a 100% yield (Table 2). 

### 3.3. Combined Effects

In order to study the influence of both temperature and sonication, a new set of Al-deposits were obtained. Three temperatures (20 °C, 40 °C, and 60 °C) and three mixing parameters (quiet bath, stirring at 320 RPM, and sonication 1 to 10) were chosen to deposit aluminium on 25 mm diameter disks. Table 3 summarizes the tested combinations, while the electrochemical set-up used is depicted in Figure 2b. The sample at 20 °C in quiet was not studied since it required a very negative potential (close to −4 V), well below the IL cathodic limit.

Deposition at high temperature combined with strong sonication duty cycle (30%, 1 to 1) is not of practical interest since the heating effects of sonication cause local temperatures to rise, which leads to quick degradation of the IL. As expected, the yield increases as a function of temperature (samples A and B) and mixing (Table 3).

**Figure 8:**
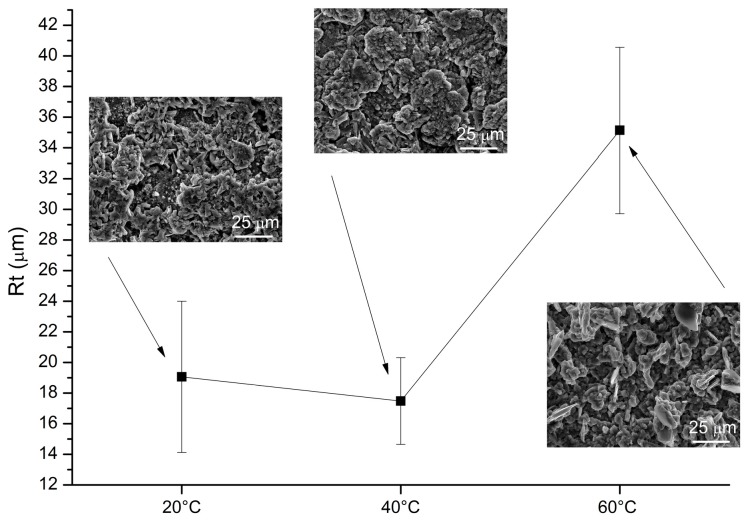
Roughness and SEM micrograph (scale bar is 25 μm) of the samples obtained by mechanical stirring (320 RPM) at different temperatures.

**Figure 9:**
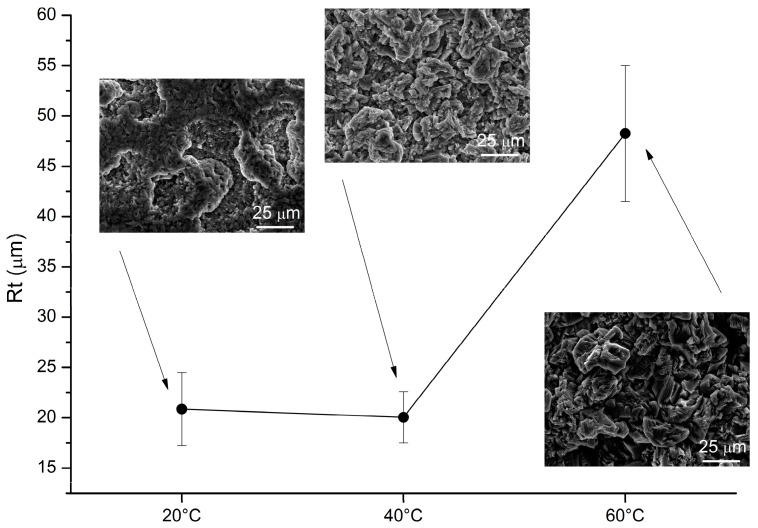
Roughness and SEM micrograph (scale bar is 25 μm) of the samples obtained via sonication (1 to 10 cycle) at different temperatures.

Roughness and morphological investigation on the Al-deposits are depicted in Figure 8 and Figure 9. A substantial increase of the crystal size of the deposits obtained at higher temperatures accounts for the observed increased roughness. 

#### Structural Investigation

**Figure 10:**
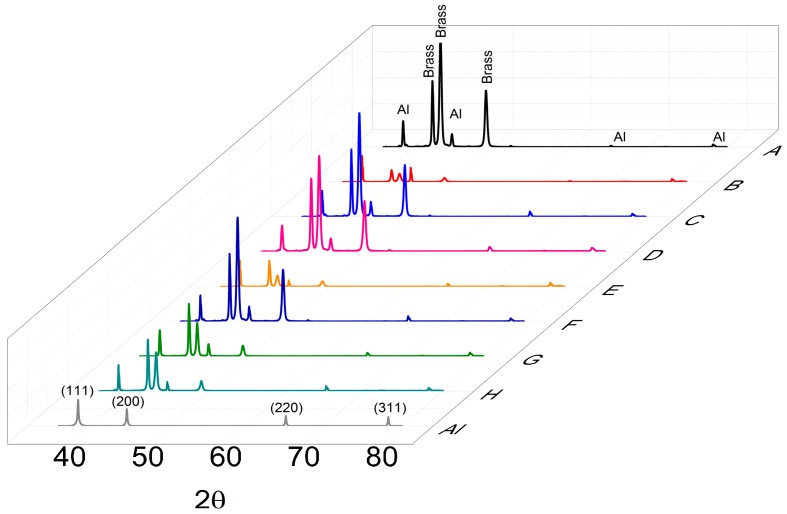
X-ray diffraction (XRD) patterns of the samples (A–H) in Table 3 compared with the simulated powder aluminium pattern (Al).

The XRD patterns of the Al-coated samples (from A to H) compared with the simulated Al metal pattern are depicted in Figure 10 (PDF 85-1327). The substrate (63% Cu, Zn 37% brass alloy) is an α–β biphasic alloy (Alloy Phase Diagrams, ASM international, page 48), the α-brass pattern is labelled as “Brass” according to the PDF 50-1333 card, while the β-phase pattern (PDF 02-1231) is hindered by the pattern of the Al coating.

**Figure 11:**
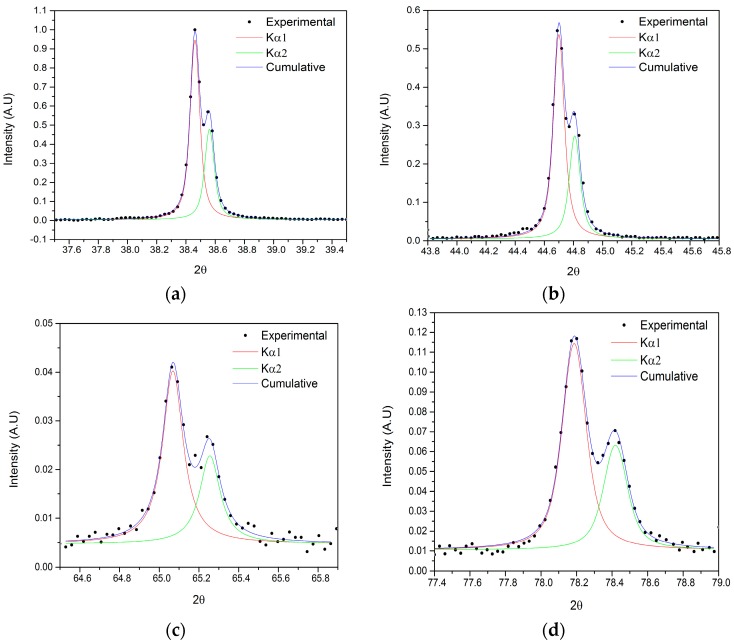
An example of the fitted double Voigt for the sample B: plane (111) (**a**); (200) (**b**); (220) (**c**); and (311) (**d**).

The diffraction peaks have been fitted with a R^2^ always higher than 0.987. Figure 11 depicts the resulting fits of the K_α1_ and K_α2_ diffraction peaks for the sample B. 

**Figure 12:**
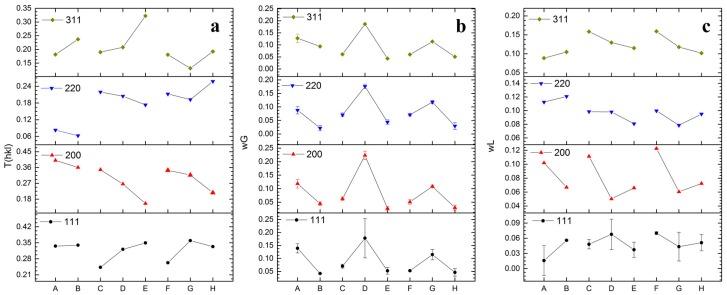
Trends of the fits for the assigned peaks over the three different sets of results: (**a**) the texture coefficient (no error bars); (**b**) the Gaussian width (proportional to the average strain); and (**c**) the Lorentzian width (proportional to the average size of the crystallites). The missing error bars in panels b and c are related to errors smaller than 0.5%.

The texture coefficients obtained from these spectra (Figure 12a) clearly show the effect of deposition parameters on the growth of the deposits. In the samples obtained by using mechanical stirring (samples C to E), the texture along (111) and (311) planes increases as a function of the temperature. On the contrary, texturing of planes (200) and (220) decrease with rising temperatures. Samples obtained by means of sonication (samples F to H) show a similar but less enhanced texturing as a function of the temperature. Moreover, the (220) plane is characterized by a significant increase of texturing for the sample obtained at 60 °C.

Figure 12b reports the Gaussian widths of the XRD peaks. These values are considered to be related to the average strain of the crystallites. It is worth noting that for each plane the maximum Gaussian width, hence the maximum lattice strain, was achieved in the deposits obtained at 40 °C. Regarding the Lorentzian width of the peaks, which is inversely related to the average crystal size, the obtained values are depicted in Figure 12c. The average crystallite size presents trends that are more complex with respect to the texture. Generally speaking, improving the mixing conditions does not dramatically affect crystallite size along (111), (200), and (220) planes. In particular, the temperature does not consistently change the size along the zonal axis of the (111) plane. Along the zonal axis corresponding to the (200) and (220) planes, the crystallites size reaches its maximum at 40 °C under sonication. On the other hand, along the zonal axis of the (311) plane, the average crystallite size increases consistently with the temperature.

## 4. Conclusions

We have successfully prepared thick coatings of metallic aluminium on brass substrates by electrodeposition from (Bmim)Cl/AlCl_3_ 40:60 ionic liquid. The investigation of the surface morphology, the crystal orientation, as well as the process yield evidenced the effects of temperature and mixing conditions. 

Particularly, the process yield proved useful to assess the optimal deposition conditions to achieve compact and dense deposits. Yield lower than 100% can be related to the formation of dendritic coatings due by mass transfer limitations. Regarding the effect of operative conditions, temperature increase plays a positive role enhancing the deposition yield and smoothing the roughness of the deposits. On the other hand, sonication greatly improves the mobility of the species inside the IL allowing 100% yield, but also forcing a slight increase in roughness [25].

The combined effects of the experiments demonstrated that for temperatures above 60 °C, mixing has a larger effect with respect to temperature in reducing the roughness of the deposits. Below 40 °C, temperature affects the morphology of the coatings more effectively. It is reasonable to suppose that the two effects have a competitive role in growing the layer, with a threshold assessed between 40 °C and 60 °C. Compared to the sonication, stirring shows a lower yield at nearly room temperatures, but at higher temperatures the morphological and roughness studies show similar trends between these two mixing systems. 

Regarding XRD analysis, the texturing of the Al coating is related to the temperature of the deposition process, favouring the orientation along the (111) and (311) axis. That is confirmed by the crystallite size analysis which demonstrated the preferential growth along different zonal axis according to different mass transport conditions [26]. In general, the mixing conditions do not affect the crystallites’ size, while increasing the temperature does. Moreover, the maximum of the crystallite strain at 40 °C, in each set of mixing conditions, can be explained as a higher concentration of defects in the crystallites. This phenomenon cannot be confirmed in this study and the reason for its occurrence has to be understood. We suggest that it can be considered a trade-off between the nucleation-growth mechanism and the improvement of the mass transport of the active species. 

In conclusion, higher temperatures and stronger mixing conditions promote the deposition of smoother and thicker aluminium deposits. However, in order to move towards the industrialization of the process and to increase the durability of the IL, temperatures above 60 °C should be avoided. In order to speed up the electrodeposition process, mechanical stirring or mild sonication can be successfully adopted.

## Figures and Tables

**Table 1:** Roughness parameters for the samples obtained at different temperatures and their relative yield.

Sample	Rt (μm)	Ra	RzISO (μm)	Rz (μm)	Yield (%)
Temp. 50 °C	32.9 ± 1.2	3.7 ± 1.1	25.5 ± 4.1	23.3 ± 2.8	79
Temp. 70 °C	21.7 ± 10.5	2.1 ± 0.2	14.9 ± 5.8	13.4 ± 4.8	86
Temp. 90 °C	4.2 ± 0.1	0.3 ± 0.1	3.1 ± 0.1	2.90 ± 0.1	88

**Table 2:** Roughness parameters for the samples obtained with different agitation modes and their relative yield.

Sample	Rt (μm)	Ra	RzISO (μm)	Rz (μm)	Yield (%)
Quiet deposit	4.2 ± 0.3	0.4 ± 0.1	3.6 ± 0.2	3.4 ± 0.2	60
Sonication 1 to 10	11.7 ± 1.3	0.7 ± 0.1	8.5 ± 0.8	7.2 ± 0.6	100
Sonication 1 to 1	17.0 ± 1.2	1.7 ± 0.1	13.6 ± 0.6	12.7 ± 0.4	100

**Table 3:** Summary of the samples obtained by means of combined experimental conditions and, within brackets, their relative process yield.

Yield %	20 °C	40 °C	60 °C
Quiet Deposits	–	A (45%)	B (69%)
Stirring 320 RPM	C (82%)	D (100%)	E (100%)
Sonication 1 to 10	F (100%)	G (100%)	H (100%)

## Abbreviations

The following abbreviations are used in this manuscript:

IL	Ionic liquid
Bmim	1-Butyl-3-methyl-imidazolium
SCE	Standard calomel electrode
WG	Gaussian width
WL	Lorentzian width
OCP	Open circuit potential
PD	potentiodynamic

## Appendix A. Roughness

Four different roughness parameters are considered in this article: Rt, Ra, Rz ISO, and Rz [27,28].

Rt is the so called total roughness, the height between the deepest valley and the highest peak extrapolated from the entire evaluation length.

Ra is the average roughness, the arithmetic mean of the profile from a mean line.

Rz ISO and Rz are similar to the Rt value; in fact, they are expressed as the mean of the sum of the maximum value of profile peak height and the maximum profile valley depth taken in various sampling lengths within the evaluation length (ten for the Rz ISO and five for the Rz).

While Ra and Rt alone may be of very limited value in assessing the overall surface roughness of a sample (particularly Rt which can be seriously affected by a single spurious scratch or particle of dirt on the surface), their conjunction with the two Rz values could greatly boost our comprehension of the homogeneity of the obtained deposits. In addition, the variance related to the roughness measurements helped us to visualize the surface homogeneity of the obtained deposits. In fact, roughness parameters were collected considering five measurements within three samples for each experiment. Larger errors are related to higher inhomogeneity of the surfaces, while smaller ones represent more homogeneous surfaces.
